# Protection Evaluation of a New Attenuated ASFV by Deletion of the *L60L* and *CD2v* Genes against Homologous Challenge

**DOI:** 10.3390/v16091464

**Published:** 2024-09-14

**Authors:** Jinjin Yang, Rongnian Zhu, Nan Li, Yanyan Zhang, Xintao Zhou, Huixian Yue, Qixuan Li, Yu Wang, Faming Miao, Teng Chen, Fei Zhang, Shoufeng Zhang, Aidong Qian, Rongliang Hu

**Affiliations:** 1 College of Veterinary Medicine, Jilin Agricultural University, Changchun 130022, China; jjyang2021@163.com (J.Y.); wangyu13558543693@163.com (Y.W.); 2 State Key Laboratory of Pathogen and Biosecurity, Key Laboratory of Jilin Province for Zoonosis Prevention and Control, Changchun Veterinary Research Institute, Chinese Academy of Agricultural Sciences, Changchun 130117, China; 18624339761@163.com (R.Z.); linan226@126.com (N.L.); yanyanzhang90615@163.com (Y.Z.); zhouxtao@foxmail.com (X.Z.); 18380384852@163.com (H.Y.); li.qi.xuan@foxmail.com (Q.L.); miaofaming81@163.com (F.M.); ctcx1991@163.com (T.C.); fei2333@163.com (F.Z.); zhangshoufeng@hotmail.com (S.Z.); 3 Key Laboratory of Prevention & Control for African Swine Fever and Other Major Pig Diseases, Ministry of Agriculture and Rural Affairs, Changchun 130117, China

**Keywords:** African swine fever, African swine fever virus, recombinant virus, deletion, live attenuated vaccine candidate

## Abstract

African swine fever (ASF) is an acute infectious disease with a high mortality rate in both domestic and wild boars. Commercial vaccines or antiviral drugs for ASF were not available due to the complex diversity of the structure and genome of its pathogen African swine fever virus (ASFV). In recent years, there have been many reports on candidate strains of attenuated vaccines for ASFV. In this study, we obtained a recombinant virus named SY18ΔL60LΔCD2v by simultaneously deleting the *L60L* gene and *CD2v* gene from highly virulent strain SY18. In vitro, SY18ΔL60LΔCD2v displayed a decreased growth kinetic compared to that of parental SY18. In vivo, high doses (10^5^ TCID_50_) of SY18ΔL60LΔCD2v can protect pigs (5/5) from attacks by the parental SY18 strain (10^2^ TCID_50_). Low doses (10^2^ TCID_50_) of SY18ΔL60LΔCD2v only protected 20% of pigs (1/5) from attacks by the parental SY18 strain (10^2^ TCID_50_). The results indicated that the absence of these two genes in SY18 could induce protection against the homologous parental strain, and there were no obvious clinical symptoms or viremia. These results indicate that the SY18ΔL60LΔCD2v strain can serve as a new live attenuated vaccine candidate for the prevention and control of ASFV infection.

## 1. Introduction

African swine fever (ASF) is an acute infectious disease caused by the African swine fever virus (ASFV), and the mortality rate of domestic pig and wild boars can reach 100% once infected. ASF is circulating in many countries in Africa, Europe, and Asia, and the lack of vaccines poses a huge challenge to the prevention and control of ASF [1,2,3]. ASF has been prevalent in East Asia and Southeast Asia since 2018, mainly with genotype II, although there have been reports of genotype I in China [4,5,6,7,8].

ASFV is a large double-stranded DNA virus, with different isolated strains ranging in length from 170–193 kbp. This variability is caused by the deletion or insertion of MGF family genes. The ASFV genome has 151–167 open reading frames, with a complex structure and diverse functions. The functions of many encoded proteins remain unclear [4,9]. At present, genes such as *I177L*, *A137R*, and *I73R* have been proven to be important virulence genes in ASFV [10,11,12,13]. In our previous research, we identified a virulence-related gene *L60L* [14]. Deletion of *L60L* from the highly virulent strain SY18 (genotype II) could significantly reduce virulence and provide protection against the parental strain [14]. The *CD2v* protein, encoded by the *EP402R* gene, is mainly involved in the blood adsorption characteristics of ASFV. *EP402R* is a virulence-related gene, and the deletion of *EP402R* from the BA71 strain can provide cross-protection against different virulence strains [15,16,17]. 

Serious damage has been caused to the global pig farming industry because of the prevalence of ASFV. Prevention and control measures have always been based on early detection, disinfection, and sterilization of infected or affected animals. In the past and even now, many studies have found that attenuated live vaccine candidate strains obtained through genetic engineering operations may be the direction of future vaccine applications [18,19,20]. The impact of different gene deletions on viruses is not the same in the process of studying vaccines. It was shown that deletion of the *L60L* gene in the highly virulent strain SY18 only partially reduces the virulence of virus and still causes viremia [14]. However, studies have reported that consecutive deletions of two or more genes can reduce viremia levels and clinical features, which can effectively improve the safety of the candidate vaccine in swine [21,22,23,24]. Therefore, it is necessary to continue deleting a virulence gene based on the existing SY18ΔL60L to reduce virus virulence and improve the safety of candidate vaccines.

In this study, a recombinant virus with continuous deletion of *L60L* and *CD2v* genes was constructed based on virulent ASFV SY18. Then, the safety and immunoprotective efficacy of the double-gene-deletion strain was further evaluated through in vivo experiments.

## 2. Materials and Methods

### 2.1. Virus and Cells

The acquisition of primary bone marrow-derived macrophages was as described in the previous research of our laboratory. The cell samples were tested for pig-derived exogenous viruses, and it was found that this batch of cells is free of exogenous virus infection [25]. The detected exogenous viruses include ASFV, classical swine fever virus (CSFV), porcine reproductive and respiratory syndrome virus (PRRSV), pseudorabies virus (PRV), porcine parvovirus (PPV), and porcine circovirus ½ (PCV1/2). Cells were cultured in RPMI 1640 (Gibco, Beijing, China) medium supplemented with 10% fetal bovine serum (Gibco) and 10 ng/mL GM-CSF. The cell culture temperature was 37 °C and the concentration of carbon dioxide was 5%. The wild-type virus, ASFV SY18 (GenBank No.: MH766894.2), was isolated by Epidemiology Laboratory of the Military Veterinary Research Institute. 

The main steps in virus titration were to infect BMDMs pre-seeded in 96-well plates with a continuous 10-fold limited dilution virus. After 4 days of cultivation in a cell culture incubator, ASFV SY18 was fixed with 80% acetone for 30 min, blocked with 5% BSA at 37 °C for 1 h, then stained with a fluorescein isothiocyanate (FITC)-labeled anti-p30 monoclonal antibody at 37 °C for 1 h, and finally observed under a fluorescence microscope. SY18ΔL60LΔCD2v was observed directly under a fluorescence microscope. The Reed–Muench method was used to calculate virus titration [26].

### 2.2. Generation of Recombinant Viruses

The recombinant virus was obtained by homologous recombination and infecting the parental strain, following the method described in previous literature [19]. The recombinant transfer vector pΔL60L-mCherry contained the left and right arms (with a length of approximately 1200 bp) of the *L60L* gene, as well as a reporter cassette p72 mCherry, which replaced the *L60L* gene. BMDMs were transfected with this recombinant plasmid and infected with the SY18 at an MOI of 1 at 6 h post-transfection. After 16 h, the virus SY18ΔL60L was purified through limited dilution. The design method of another recombinant vector pΔCD2v-EGFP was the same, except that the expression cassette was replaced with p72 EGFP. Finally, BMDMs were transfected with this recombinant plasmid and infected with the SY18ΔL60L at an MOI of 1 at 6 h post-transfection. The double-gene-deletion virus SY18ΔL60LΔCD2v was identified after several rounds of fluorescence microscopy observation and limited dilution purification.

### 2.3. PCR Confirmation and Next-Generation Sequencing

The recombinant virus was identified by PCR to determine complete deletion of *L60L* and *CD2v* genes from the genome of the SY18 strain. Primers were designed for the *L60L* and *CD2v* genes. Two pairs of specific identification primers were as follows: 60-I-F: TTGATGATTCAGTATTTTGTG, 60-I-R: TCCTAAACAGATGACTCCAAC. CD2v-I-F: GCAGGTAACTTTTGTGAATG, CD2v-I-R: GCTGTAAAATTGTTAATGTTAC. To verify the accuracy of the recombinant virus genome, the genome of the recombinant virus was extracted for next-generation sequencing. Sequencing was performed using Illumia NovaSeq 6000, PE150 (Novogene Co., Ltd., Tianjin, China).

### 2.4. The Growth Characteristics of Recombinant Viruses In Vitro

To compare the growth characteristics of recombinant viruses in vitro with the parental strain, BMDMs pre-seeded in 24-well plates were infected with ASFV SY18 and the recombinant virus (SY18ΔL60LΔCD2v) at an MOI of 0.01. Samples were collected at 2, 12, 24, 48, 72, and 96 h post-infection (hpi) and subjected to 3 repeated cycles of freezing and thawing. Finally, the virus titer was uniformly measured and calculated by endpoint dilution method.

### 2.5. Animal Experiments

Animal experiments were carried out in the animal bio-safety level 3 (ABSL-3) laboratory. Experiments were approved by the Animal Welfare and Ethics Committee of the Changchun Veterinary Research Institute, Chinese Academy of Agriculture (Review ID: IACUC of CAS-12-2021-011, approved on 1 December 2021).

Pigs weighing 15–20 kg were purchased from local farms with good hygiene conditions, and the qPCR method was used to detect exogenous viruses including ASFV, CSFV, PRRSV, PRV, PPV, and PCV1/2 from relevant pig sources, which were negative. Fifteen pigs were divided into three groups. Group A (numbers 30, 31, 32, 89, and 93) and group B (numbers 33, 34, 35, 36 and 37) were inoculated intramuscularly with 10^2^ and 10^5^ TCID_50_ of the recombinant virus per pig, respectively, while pigs in group C (numbers 01, 02, 03, 04, and 05) were used as blank controls until the 28th day for the challenge experiment. During the 28-day immune observation period, the rectal temperature of immunized pigs was measured daily to observe whether there were clinical symptoms such as anorexia during feeding. Heparinized whole blood, serum, oral swabs, and anal swabs were collected on days 0, 3, 7, 14, 21, and 28 of immunity, and the viral genome level was detected using qPCR methods. Immune observation was conducted for 28 days, and a challenge test was conducted on surviving pigs from Group A and Group B, using SY18 at a dose of 10^2^ TCID_50_/pig. Similarly, the rectal temperature of pigs was tested daily during the virus attack, and blood, serum, oral swabs, and anal swabs were collected on days 0, 3, 7, 14, and 21 after the challenge to detect viremia and virus shedding via qPCR. Finally, the pathological changes of various tissues and organs were analyzed through tissue pathological sections, including the liver, spleen, lungs, kidneys, and submandibular lymphoid tissue.

### 2.6. Quantitative PCR

The virus genome was extracted from collected blood and tissue samples for quantitative detection. The target gene of real-time quantitative PCR was the p72 gene of ASFV, which was developed and used by our laboratory. The specific operating procedure was also as described earlier [25].

### 2.7. Detection of Anti-ASFV Antibodies

Immunogenicity studies were conducted on pig serum collected during the immunization and challenge period. An indirect ELISA kit (developed by our laboratory) targeting the p54 protein antigen was used to detect antibodies against ASFV p54. The measured optical density value was set to OD450. The ratio of the optical density value of the measured sample to the optical density value of the positive sample was the S/*p* value. The cut-off value was 0.25 and samples were considered positive when S/*p* > 0.25 and negative when S/*p* ≤ 0.25.

### 2.8. Statistical Analysis

Statistical significance was determined using unpaired, double-tailed, and *t*-tests, and results at a *p*-value < 0.05 were considered significant.

## 3. Results

### 3.1. Generation and Identification of a Recombinant Virus SY18∆L60L∆CD2v

**Figure 1:**
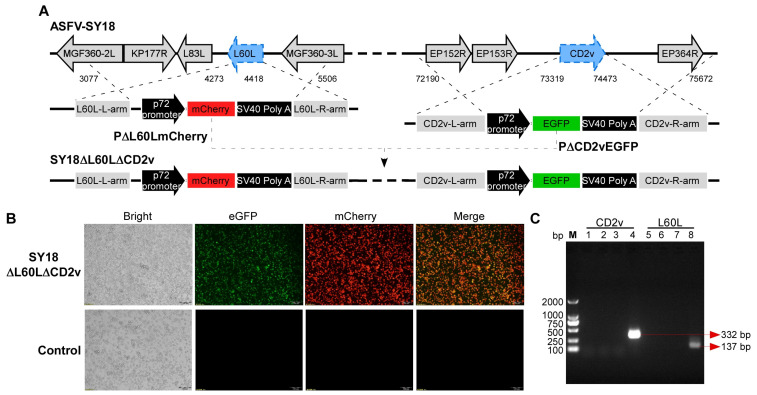
Identification and characterization of the recombinant SY18∆L60L∆CD2v. (**A**) Schematic diagram of the genome structure of the recombinant virus SY18∆L60L∆CD2v. (**B**) SY18∆L60L∆CD2v amplification in BMDMs. SY18∆L60L∆CD2v or uninfected BMDMs are shown with a different fluorescence background (100×). (**C**) Identification of the gene deletion in SY18∆L60L∆CD2v. Viral DNA from SY18∆L60L∆CD2v-infected BMDMs were tested for the presence of CD2v or L60L genes (lanes 1, 2, 5, and 6 are samples of BMDMs infected with SY18∆L60L∆CD2v; lanes 3 and 7 are samples of uninfected BMDMs; lanes 4 and 8 are samples of BMDMs infected with SY18).

The deletion of the *L60L* gene and *CD2v* gene from the SY18 high virulence strain was achieved through homologous recombination, where the two deletion genes were replaced by expression cassettes with the ASFV *p72* gene promoter, p72-mCherry, and p72-EGFP, respectively (Figure 1A). The recombinant virus was purified through eight rounds of observation under a fluorescence microscope and limited dilution on cells (Figure 1B). Primers targeting the *L60L* gene and *CD2v* gene of SY18 were designed and the final purification of the recombinant virus was identified by amplifying the PCR products of the recombinant virus and SY18. The final identification of the recombinant virus without parental strain SY18 contamination was achieved by PCR (Figure 1C).

To evaluate the accuracy of the recombinant virus genome, next-generation sequencing was performed on the entire genome of the recombinant virus. Compared with the wild-type virus, the sequencing results accurately confirmed the deletion of the *L60L* gene and *CD2v* gene, and the entire genome showed almost no significant changes.

### 3.2. Growth Characteristics of Recombinant Viruses in BMDMs

**Figure 2:**
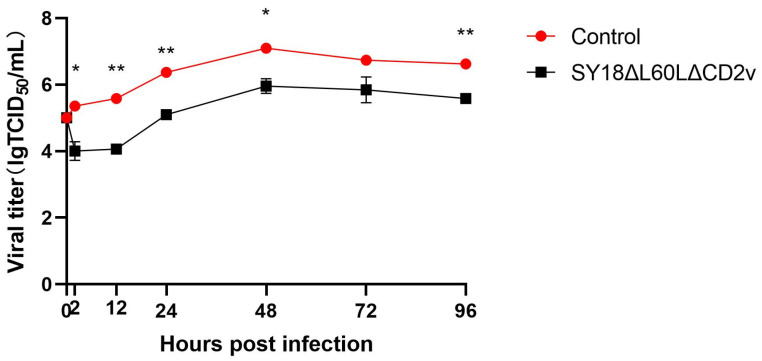
The growth characteristics of SY18 and SY18∆L60L∆CD2v in vitro. BMDMs were infected with SY18 and recombinant viruses SY18∆L60L∆CD2v at a MOI of 0.01. Samples were collected at different times, and then subjected to three freeze–thaw cycles. Finally, virus titers were measured through titration and the data are represented as the average of three sets of data and expressed as lgTCID_50_/mL. * *p* < 0.05, ** *p* < 0.01.

BMDMs were infected with ASFV SY18 and SY18∆L60L∆CD2v at a MOI of 0.01. The cell cultures were then collected at different time points. The growth curve was plotted using virus titers at different times. The data represent the results of three independent experiments. It was found that the viral titers of recombinant viruses with the deletion of the *L60L* and *CD2v* genes in BMDMs were 10 times lower than those of wild-type viruses, which is consistent with the results of the single deletion of the *L60L* gene (Figure 2).

### 3.3. Research on the Virulence and Immune Protection of Recombinant Virus in Pigs

**Figure 3:**
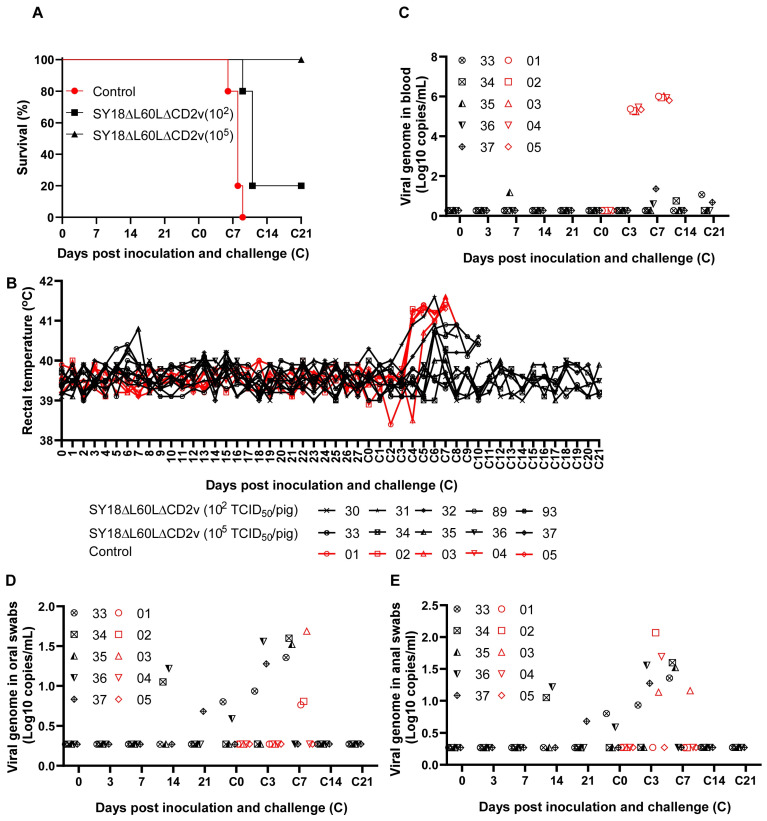
Virulence and immune protection of recombinant virus in pigs. (**A**) Survival rate of pigs infected with the recombinant virus SY18∆L60L∆CD2v and parental virus SY18. (**B**) The rectal temperature of all pigs during the entire experimental observation period. (**C**) The virus load of blood was detected in pigs immunized with high-dose SY18∆L60L∆CD2v and challenged with SY18. The viral load in oral (**D**) and anal swabs (**E**) was determined in pigs. The value is expressed in 10 copies per milliliter of log.

To study the virulence of SY18∆L60L∆CD2v in pigs, 15 pigs weighing 15–20 kg were randomly divided into 3 groups. Group A (numbers 30, 31, 32, 89, and 93, *n* = 5) and group B (numbers 33, 34, 35, 36, and 37, *n* = 5) were inoculated intramuscularly with 10^2^ and 10^5^ TCID_50_ of the recombinant virus per pig, respectively. Pigs in group C (numbers 01, 02, 03, 04, and 05, *n* = 5) were intramuscularly injected with 1 mL of physiological saline per head to be used as blank controls. Blood, oral swabs, and anal swabs were collected during the 28-day observation period. The results showed that no clinical symptoms related to ASF were found and all pigs maintained a normal body temperature and health status (Figure 3A,B) during the 28-day monitoring period. 

To evaluate whether pigs immunized with SY18∆L60L∆CD2v can resist homologous virus challenge, 15 surviving pigs were intramuscularly injected with 10^2^ TCID_50_ ASFV SY18 at 28 days post-inoculation (dpi). Simultaneously, five pigs that were not immunized with any substance were inoculated with ASFV SY18 of 10^2^TCID_50_. As a result, pigs immunized with a high dose of SY18∆L60L∆CD2v were provided complete protection against a virulent challenge of ASFV SY18 at 21 days post-challenge (dpc), while all pigs inoculated with ASFV SY18 died from the disease (Figure 3A,B). 

To evaluate the viral replication level of surviving pigs after an ASFV SY18 challenge, the viral genome in blood, oral swabs, and anal swabs of pigs was collected at different times and quantified via qPCR. The results showed that pigs vaccinated with a high dose of inoculated recombinant virus and which survived after being challenged with SY18 displayed low levels of virus replication. There was an extremely low level of the virus genome in pig blood, oral swabs, and anal swabs (<10^2^ copies/mL, Figure 3C–E), and almost no virus was detected in pigs immunized at high doses; that is, pigs immunized with high doses of the virus rarely developed detectable viremia.

### 3.4. Antibody Production in Pigs Immunized with a High Dose

**Figure 4:**
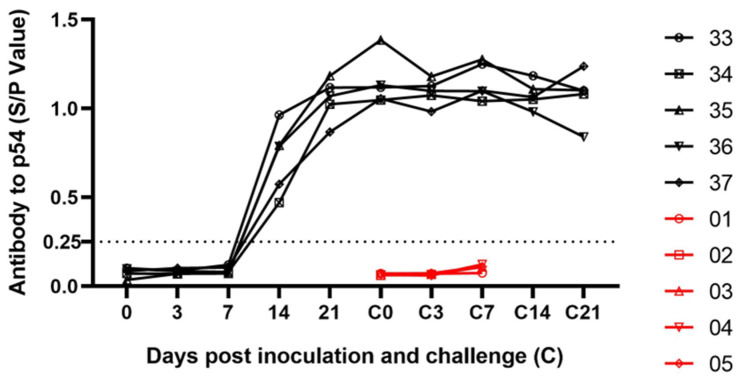
Antibody levels in pig serum. The specific antibody levels in pig serum were detected by an ASFV indirect ELISA kit.

Similarly, the indirect enzyme-linked immunosorbent assay (ELISA) was used to detect the level of anti-ASFVp54 specific antibodies in immunized high-dose pig serum (Figure 4). The antibody level started to increase from day 7 and remained at a high level until day 28. In contrast, pigs in the control group did not develop high antibody levels until they died. In summary, the double-gene-deletion strain SY18∆L60L∆CD2v induced anti-ASFV-p54-specific antibodies, resulting in a significant level of humoral immune response, indicating that it had a good immunogenicity in the high-dose immunization group.

### 3.5. Determination of Viral Genome Content in Various Tissues and Organs of Immune Pigs after Autopsy

**Figure 5:**
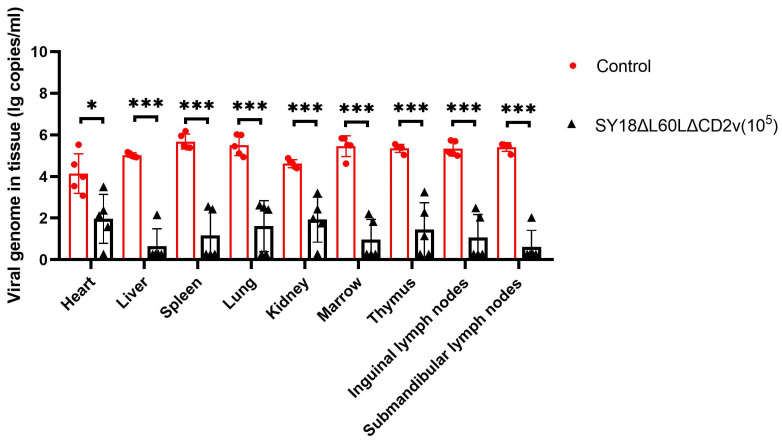
Virus distribution in tissues, organs, and lymphoid tissues of pigs immunized with high doses. * *p* < 0.05, *** *p* < 0.001.

To verify the safety of vaccine candidate strains, pigs in the high-dose immunization group were euthanized after 21 days of challenge with SY18. The heart, liver, spleen, lung, kidney, and submandibular lymphoid tissues were taken separately, and the genome was extracted from the homogenate of the tissue organs for quantification via qPCR. Similarly, the viral genome was extracted from the tissues and organs of pigs that died from the challenge with SY18, and the viral genome content was quantified. The results showed that pigs that survived high doses of immunity had a lower virus content in their bodies, which was also the reason why they could be protected against the homologous virus SY18 and survive (Figure 5).

### 3.6. Piglets Immunized with a High-Dose SY18∆L60L∆CD2v Are Protected, as Shown by Postmortem and Histological Analysis

**Figure 6:**
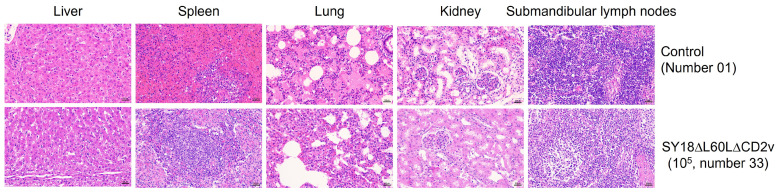
Histopathological analysis of liver, spleen, lung, kidney and submandibular lymph nodes in pigs immunized with a high dose of SY18∆L60L∆CD2v and control (200×).

To evaluate the pathological changes in various tissues and organs of piglets immunized with the high-dose SY18∆L60L∆CD2v strain after 21 days of infection with SY18, pathological sections of Group B (number 33, labeled with SY18∆L60L∆CD2v in the legend) and Group C (number 01, labeled with Control in the legend) were examined in the livers, spleens, lungs, kidneys, and lymph nodes. No significant pathological changes were found in the tissues and organs of pigs that survived the high-dose SY18∆L60L∆CD2v virus challenge compared to pigs that were challenged by the wild-type virus SY18 (Figure 6).

## 4. Discussion

The 150 proteins encoded by the ASFV genome play different roles at various stages of its lifecycle, but there are still many genes whose functions remain unclear so far [27]. Understanding the different gene functions of ASFV is crucial for developing effective vaccines and antiviral drugs. Many gene’s functions have been revealed, especially some virulence-related genes, which can significantly reduce the virulence of the virus in pigs and resist challenge from the parental strain after deletion [10,13]. Moreover, the combined deletion of multiple genes from highly virulent strains can also provide pigs with safe and effective immunity to resist attacks from the parental strain. For example, the recombinant virus obtained by simultaneously deleting the 9GL and UK genes from the genome of the Georgia 2007/1 strain conferred strong protection to pigs against homologous challenge [28]. The recombinant virus obtained by deleting *9GL* and *MGF360*/*MGF505* from the genome of the Georgia 2007/1 strain attenuated its virulence in pigs but could not protect against homologous challenge [29]. The recombinant virus obtained by deleting the *MGF360* and *MGF505* genes from the genome of the Georgia 2007/1 strain attenuated the virus’s virulence in pigs and provided protection against homologous virus attacks [19]. *CD2v* is a known virulence gene that affects the blood adsorption characteristics of the virus. The simultaneous deletion of *CD2v* and *UK* genes from highly virulent virus strains can weaken the virus’s virulence in pigs, and immunization with recombinant virus 10^4^TCID_50_ can provide challenge protection [30]. However, the recombinant virus obtained by simultaneously deleting *CD2v* and *A238L* genes from the genomes of other strains was partially attenuated in pigs [31]. Different vaccination methods and immune doses were also important for improving immune protection efficiency, which would be also a future research direction [32].

Based on our previous studies, deleting the *L60L* gene from ASFV SY18 reduces the virulence of the virus in pigs, but only 60% of pigs survived after they were immunized with a high-dose SY18∆L60L strain [14]. Therefore, this study aims to reduce virus virulence and improve the safety of vaccine candidate strains by further deleting another virulence gene, *CD2v*. Fortunately, all pigs immunized with the high-dose dual-gene-deletion viruses survived and were able to resist attacks from their parental strain without significant pathological damage to various tissues and organs after challenge. This result indicates that the combined deletion of the *L60L* and *CD2v* genes does indeed weaken the virulence of the virus. The efficiency of immune protection is a critical factor in developing attenuated live vaccines against ASFV through genetic engineering. In this study, piglets were vaccinated with the recombinant virus by intramuscular injection, and all pigs remained healthy during the 28-day observation period. Pigs in the high-dose immunization group were able to be completely protected against homologous virus challenges. Additionally, surviving pigs showed high levels of antibodies against ASFV p54. Pathological examination revealed no changes in the liver, spleen, lung, kidney, and submandibular lymph nodes of all surviving pigs. These results indicate that SY18∆L60L∆CD2v has protective effects and is safe.

As a vaccine for preventing and controlling ASF, the safety of the vaccine is a crucial consideration. Viremia, which reflects the replication and transmission of viruses in vivo, is an important factor used to assess the vaccine’s safety. Compared to pigs challenged with wild-type viruses, pigs immunized with SY18∆L60L∆CD2v showed very low or undetectable levels of the virus genome in their blood. The viremia level of SY18∆L60L∆CD2v was lower than 1 × 10^2^ copies/mL, whereas the challenge group reached 1 × 10^6^ copies/mL. In summary, this study developed a new recombinant virus by deleting the *L60L* and *CD2v* genes from the ASFV SY18 strain. The recombinant virus exhibits reduced virulence in pigs and can protect against the parental virus when used to immunize pigs with a high dose. The recombinant virus shows potential as a live attenuated vaccine candidate. Our future experimental research aims to further improve the efficacy and safety of live attenuated candidate vaccines by continuously deleting one or more virulence genes based on this foundation.

## 5. Conclusions

In this study, a recombinant virus, SY18∆L60L∆CD2v, in which the *L60L* gene and *CD2v* gene were continuously deleted from the genome of the SY18 strain, was developed. Immunization of pigs with SY18∆L60L∆CD2v completely attenuated the virulence of the virus in vivo. Pigs immunized with a high dose of SY18∆L60L∆CD2v were completely protected from challenges from the parental strain SY18. Additionally, surviving pigs produced specific antibodies against the ASFV p54 antigen. Pigs that were immunized with a high-dose virus and survived after challenge with SY18 did not show significant pathological changes in various tissues and organs. Therefore, this recombinant virus holds significant research potential as a candidate for live attenuated vaccine development.

## Data Availability

The original contributions presented in the study are included in the article.
